# Evaluation of the accuracy of intraoral scanners for nasal digitization: an *in vitro* three-dimensional comparative study

**DOI:** 10.3389/froh.2025.1678778

**Published:** 2025-11-07

**Authors:** Mariko Hattori, Sandra Stadler, Ralf J. Kohal, Kirstin Vach, Mahmoud E. Elbashti, Yuka I. Sumita, Noriyuki Wakabayashi

**Affiliations:** 1 Department of Advanced Prosthodontics, Division of Oral Health Sciences, Graduate School of Medical and Dental Sciences, Institute of Science Tokyo, Tokyo, Japan; 2 Private Dental Clinic, Burglengenfeld, Germany; 3 Medical Center—University of Freiburg, Center for Dental Medicine, Department of Prosthetic Dentistry, Faculty of Medicine, University of Freiburg, Freiburg im Breisgau, Germany; 4 Medical Center—University of Freiburg, Institute for Medical Biometry and Statistics, Faculty of Medicine, University of Freiburg, Freiburg im Breisgau, Germany; 5 Faculty of Dentistry, University of Zawia, Zawia, Libya; 6 Department of Partial and Complete Denture, School of Life Dentistry at Tokyo, The Nippon Dental University, Tokyo, Japan

**Keywords:** intraoral scanner, facial scanning, maxillofacial prosthetics, nasal digitization, prosthodontics

## Abstract

Taking a facial impression is essential in maxillofacial prosthetics and in dentistry when designing a dental prosthesis. This study explores the use of intraoral scanners as an alternative method for recording the surface of the face, specifically the nose. A soft head model was scanned using three different intraoral scanners, and the accuracy with which they captured the nose was compared with that obtained by conventional impressions. Two of the three scanners successfully captured the nose, demonstrating trueness and precision superior to that of conventional impressions. Intraoral scanners are a viable option for capturing the surface of the nose.

## 1 Introduction

Maxillofacial prostheses are used for rehabilitation after resection of head and neck cancer. Facial impressions are obtained using dental impression materials when creating a maxillofacial prosthesis (1). However, conventional impression techniques present several challenges. The process is often complex and uncomfortable for the patient, particularly when performed in a supine position, where the weight of the impression material and the altered direction of gravity can increase distress. Furthermore, there is a risk of foreign body aspiration or suffocation during the procedure.

Use of a facial scanner to obtain a digital impression is an alternative approach (2–4), and several types of facial scanners have been compared for accuracy (5). Nowadays, even facial expressions can be captured by facial scanners (6). However, such a device is not always available in dental clinics, whereas intraoral scanners are now widely used in routine dental practice.

Our previous research has demonstrated the accuracy of intraoral scanners for digitizing an ear model and their superior precision compared with conventional impression techniques (7). However, despite the increasing use of facial scanners, there is little evidence regarding the feasibility of using intraoral scanners for capturing nasal morphology. To our knowledge, no previous study has systematically evaluated the accuracy of intraoral scanners in this application. Given these promising findings, we considered it worthwhile to explore whether intraoral scanners could also be used for accurate capture of nasal morphology.

Facial morphology, including the lips, nose, eyes, and forehead, is often assessed when creating a prosthesis to determine the occlusal plane and midline. This would provide a practical and accessible alternative to specialized facial scanners, reducing patient discomfort and procedural risks while leveraging a device that is already widely available in dental practice. If intraoral scanners can accurately capture nasal morphology, they could be useful for fabrication of both maxillofacial and dental prostheses. Therefore, evaluation of the value of intraoral scanners for nasal digitization is of significant clinical interest. This *in vitro* study aimed to assess the accuracy of intraoral scanners when used for nasal digitization by comparing their performance with that of conventional scanning methods. According to ISO 5725-1:2023, accuracy was evaluated in terms of both trueness (closeness to the reference) and precision (reproducibility between repeated scans), which are distinct but complementary components of accuracy (8). The null hypothesis was that there would be no significant difference in the accuracy of nasal impressions between intraoral scanners and conventional impression methods.

## 2 Materials and methods

**Figure 1:**
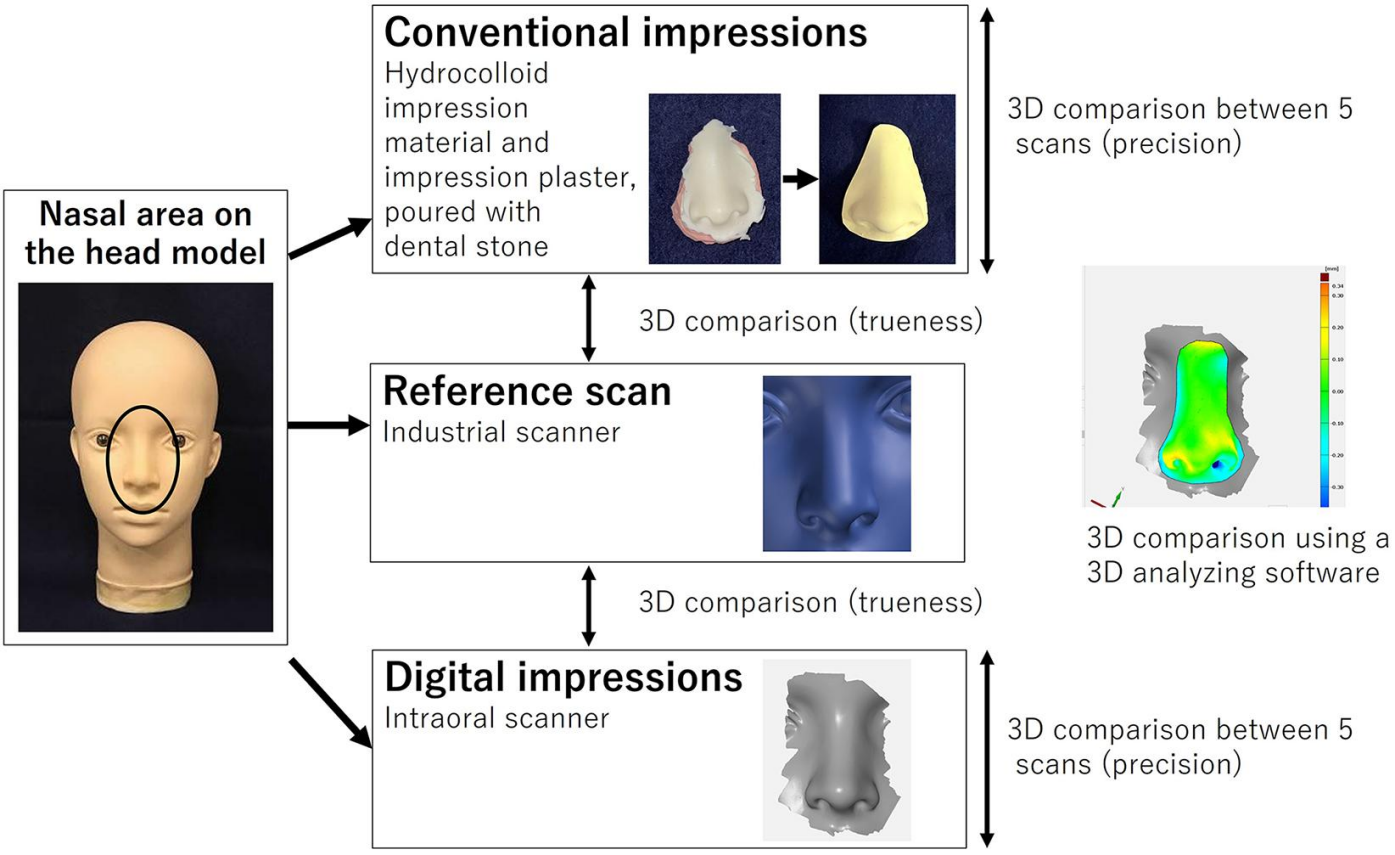
Diagram showing the data acquisition process.

A skin-colored soft head model (PVC Female Mannequin Head #3, Vococal Technology, Shenzhen, China) (Figure 1) was scanned using an industrial scanner (Atos III Triple Scan 8MP, GOM, Braunschweig, Germany). The scanner was selected as the reference standard because of its high accuracy and reliability in 3D measurements, and it has been widely adopted as a standard in previous studies (7, 9–11). Three intraoral scanners (CEREC AC Omnicam, Dentsply Sirona, Charlotte, NC, USA; True Definition Scanner, 3M ESPE, Saint Paul, MN, USA; and Cara Trios-3, Heraeus, Hanau, Germany) were then used as test groups. The nose of the model was scanned with each intraoral scanner using a circular motion. Five scans were performed with each scanner according to the manufacturer's instructions. To avoid operator-related variability, all scanning procedures were conducted by one operator. All scans were taken in the same room excluding the influence of extraneous light and under the same temperature and humidity conditions (mean temperature 22 ± 1 °C; relative humidity 45% ± 5%). Conventional impressions were also taken five times using irreversible hydrocolloid impression material (Algiace Z, Dentsply-Sankin K.K., Tokyo, Japan) and impression plaster (Xanthano, Heraeus Kulzer Dental, Shanghai, China) and poured using type III dental stone (Pico-crema soft, Picodent, Wipperfürth, Germany). The stone models were then scanned using a desktop dental scanner (i/s/can, Organical CAD/CAM, Berlin, Germany). The data files obtained were loaded into 3D inspection software (Geomagic Control X 2017, Geomagic, Morrisville, NC, USA). After cropping the unnecessary parts, the datasets were superimposed onto the reference data using a best-fit algorithm for trueness analysis. For precision analysis, all possible pairs were generated from the five scans (_5_C_2_ = 10 pairs) and were also compared using the best-fit algorithm. For both analyses, the software calculated the total absolute 3D deviations, defined as the deviation of the closest point-to-point distances. Trueness and precision values were summarized as the mean ± standard deviation. Linear mixed models with random intercepts were fitted for each sample. The method of Scheffé was used to correct for multiple testing. All statistical analyses were performed using STATA v15.1 software (StataCorp, College Station, TX, USA). The alpha level was set at 0.05 to determine statistical significance.

## 3 Results

**Figure 2:**
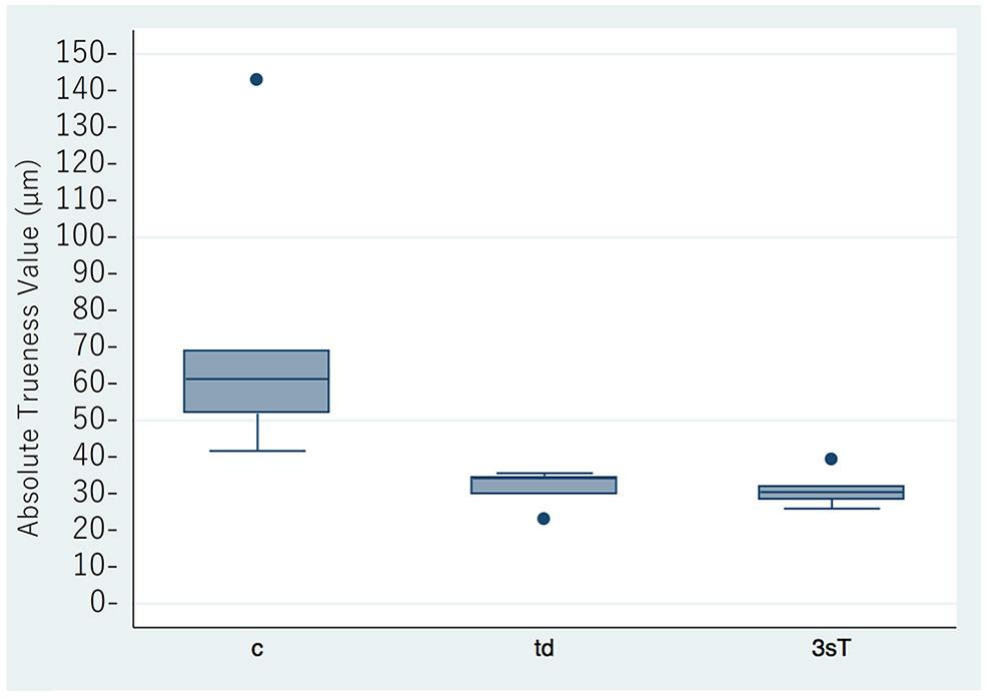
Mean absolute trueness values for the conventional and digital impressions (μm). c, conventional impression; td, true definition scanner; 3sT, Cara Trios-3 scanner.

**Figure 3:**
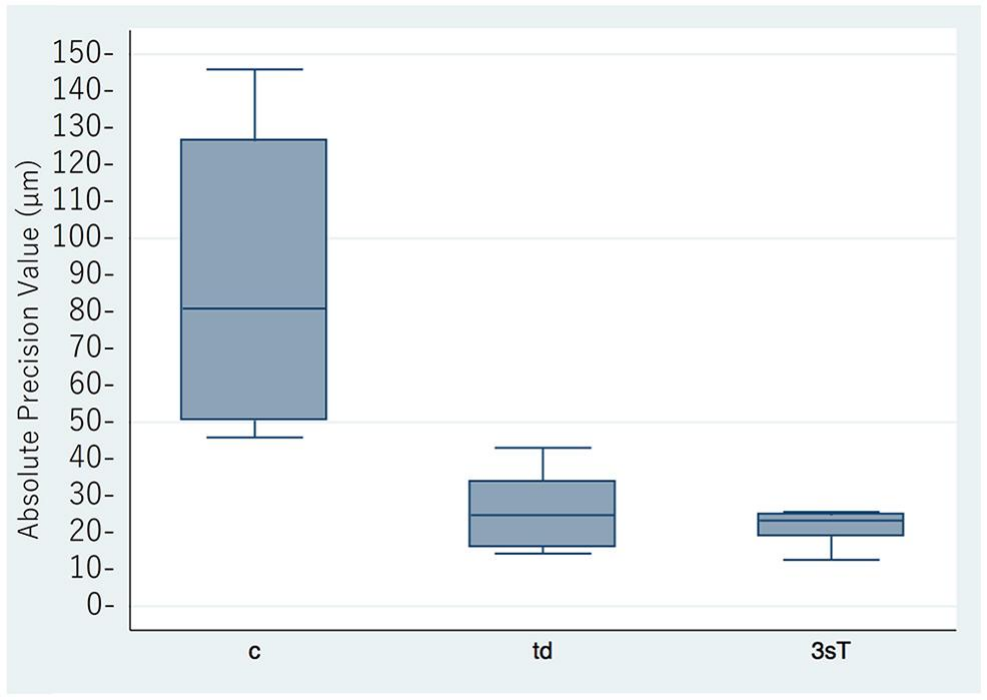
Mean absolute precision values for the conventional and digital impressions (μm). c, conventional impression; td, true definition scanner; 3sT, Cara Trios-3 scanner.

Scanning was successful with the True Definition and Cara Trios-3 scanners. Scanning stopped frequently when using the CEREC scanner, and no images were obtained. Therefore, the data for the CEREC scanner were not included in the analysis. The results for trueness and precision are shown in Figures 2,3, with details summarized in Table 1. The mean trueness values were 73.3 ± 40.2 μm (95% CI: 23.4–123.2) for the conventional impressions, 31.4 ± 5.19 μm (95% CI: 25.0–37.9) for the True Definition, and 31.2 ± 5.04 μm (95% CI: 24.9–37.4) for the Cara Trios-3. The corresponding mean precision values were 89.4 ± 38.6 μm (95% CI: 61.8–117.0), 26.6 ± 9.96 μm (95% CI: 19.5–33.8), and 21.5 ± 4.39 μm (95% CI: 18.3–24.6), respectively. Statistically significant differences in trueness and precision were found between the conventional and digital impressions (*P* = 0.007 and *P* < 0.001, respectively between conventional and True Definition, and *P* = 0.007 and *P* < 0.001, respectively between conventional and Cara Trios-3) but not between the two digital impressions (*P* = 1.000 and *P* = 0.872, respectively). The significantly smaller mean trueness and precision values for both intraoral scanners in comparison with the values obtained by the conventional method confirmed that the impressions made by the scanners were more accurate.

**Table 1:** Trueness and precision of different scanning methods (μm).

		Value (μm)			
		Trueness		Precision	
		Mean ± SD	95% CI	Mean ± SD	95% CI
Impression	c	73.3 ± 40.2	23.4-123.2	89.4 ± 38.6	61.8–117.0
	td	31.4 ± 5.19	25.0-37.9	26.6 ± 9.96	19.5–33.7
	3sT	31.2 ± 5.04	24.9-37.5	21.5 ± 4.39	18.4–24.6

c, conventional impression; td, True Definition scanner; 3sT, Cara Trios-3 scanner.

## 4 Discussion

Accurate digitization of nasal morphology is important for creation of both maxillofacial and dental prostheses (3, 6, 12–15). This study evaluated the accuracy of intraoral scanners when used for nasal digitization by comparing their performance with that of conventional impression methods. We found a significant difference in accuracy between the two approaches, leading to rejection of the null hypothesis. This finding suggests that intraoral scanners can serve as a reliable alternative for nasal digitization and offer a readily available and clinically practical solution in dental settings.

In this study, three types of intraoral scanner were used to digitize a nose model, and it was found that two scanners could capture the nose, whereas one could not. The intraoral scanners were selected because they are widely used in clinical practice and are commercially available internationally. The CEREC scanner was interrupted during the attempt to scan the nasal model. This may be attributed to the fact that the system was primarily developed for single-crown or fixed prosthesis applications rather than for capturing mucosal surfaces in denture fabrication, and it is designed to stop when encountering large flat areas. Using other two scanners, the results for accuracy were comparable with those previously reported for models of the jaw (11) and ear (7), although a nose has a smoother surface with fewer landmarks. The skin color and skin-like surface of the model could explain the accuracy of scanning in this study, considering the known difficulties of scanning white or shiny matter. The two suitable scanners differ in their impression system, light source, and imaging technology. The True Definition scanner utilizes active wavefront sampling with pulsating blue light and records 3D data as motion video, whereas the Cara TRIOS-3 scanner employs optical sectioning with a blue LED light and video-based capture (16). In addition, the requirement for scanning powder with the True Definition scanner may also influence performance. However, in the present study, no significant differences were observed between the two scanners, and therefore it is not possible to conclude which system performs better.

The findings of our present study confirm the possibility of scanning the nose using an intraoral scanner before maxillofacial surgery to create a facial prosthesis. In cases where a nasal defect remains after surgery, scanning the residual structure could provide data for prosthesis fabrication. Moreover, when the patient is referred after surgery, scanning the nose of a family member with a similar nasal morphology could provide a reference for the prosthesis (17, 18).

From a prosthodontic point of view, scanning the nose with an intraoral scanner when a new denture is fashioned might help with tooth alignment as one of the production steps (12–15). Data for both the intraoral condition and the surface of the face may help the dentist to design the prosthesis and inform the dental technician about the positional relationship between the teeth and the facial surface. The degree of accuracy observed in this study suggests that scanning of the face using an intraoral scanner will become commonplace for a variety of prosthodontic applications in the near future.

This study has some limitations. First, the *in vitro* nature of the research may not fully replicate the complexities of scanning living human skin, which varies in texture, color, and reflectivity. Second, only three types of intraoral scanner were investigated, and there may be differences in performance across other models and manufacturers. Further research is needed to assess the efficacy of intraoral scanners in clinical settings in actual patients under real-world conditions. In addition, future studies should directly compare intraoral scanners with dedicated facial scanners to provide more comprehensive insights into the relative advantages and limitations of different approaches for nasal digitization. Exploration of advanced scanning technologies or software enhancements to improve the accuracy of digitization of smooth surfaces, such as the nose, would also be useful. Finally, investigating the impact of skin tone and surface texture on scanning performance could provide valuable insights for optimizing the maxillofacial applications of intraoral scanners.

## 5 Conclusion

This *in vitro* study demonstrates that intraoral scanners can accurately capture nasal morphology, with two of the three scanners evaluated showing superior trueness and precision compared to conventional impressions. These findings indicate that intraoral scanners are a practical and clinically applicable option for nasal digitization, which may support the fabrication of maxillofacial and dental prostheses. Further studies in clinical settings are warranted to confirm these results and to explore comparisons with dedicated facial scanners, as well as the impact of skin tone, surface texture, and advanced scanning technologies on accuracy.

## Data Availability

The raw data supporting the conclusions of this article will be made available by the authors, without undue reservation.
